# Optimisation of Medicine Compounding Using Quality by Design Approach: Case Studies of Two Aqueous Cream Formulations

**DOI:** 10.3390/pharmaceutics17091232

**Published:** 2025-09-22

**Authors:** Okhee Yoo, Wenting Li, Siyu Ruan, Elizabeth Syme, Alisha Rodrigo, Connelia Locher, Sharmin Sultana, Lee Yong Lim

**Affiliations:** 1 Department of Pharmacy, School of Health and Clinical Sciences, University of Western Australia, Perth, WA 6009, Australia; okhee.yoo@uwa.edu.au (O.Y.); connie.locher@uwa.edu.au (C.L.); sharmin.sultana@uwa.edu.au (S.S.); 2 Institute for Paediatric Perioperative Excellence, The University of Western Australia, Perth, WA 6009, Australia; 3 Centre for Optimisation of Medicines, School of Health and Clinical Sciences, University of Western Australia, Perth, WA 6009, Australia; 4 Wesfarmers Centre for Vaccines and Infectious Diseases, The Kids Research Institute, Nedlands, WA 6009, Australia

**Keywords:** aqueous cream APF, cetomacrogol cream APF, quality attributes, quality by design, design of experiments, response surface model, factorial design

## Abstract

**Background/Objectives**: Quality-by-Design (QbD) is a proactive, risk-based, regulatory-endorsed approach to the development and manufacture of medicinal products but is rarely applied to medicines compounded by pharmacists. This study aims to apply the QbD approach to optimise the compounding processes for the aqueous cream and cetomacrogol cream formulations listed in the Australian Pharmaceutical Formulary and Handbook (APF). **Methods**: The creams were prepared by varying the process conditions, including oil and water phase temperatures, stirring speed, cooling environment temperature, and the temperature at the end of stirring. Thirty-two samples of each cream type were prepared using combinations of processing conditions defined by a three-level factorial design. The viscosity, spreadability and creaming index of samples were assessed as response variables, and results were analysed using Stat-Ease 360© software to determine the optimal processing conditions for the two creams. To validate the predictive model and assess further cream stability, triplicate creams of each formulation were prepared using the optimised conditions and evaluated for dynamic viscosity, spreadability and creaming index. **Results**: Optimal conditions for aqueous cream involved heating the oil and water phases to 60 °C and 80 °C, respectively, followed by stirring the two phases at 250 rpm at 10 °C until cooling to 50 °C. For cetomacrogol cream, optimal compounding required heating the oil and water phases to 70 °C and 75 °C, respectively, with stirring the two phases at 220 rpm at ambient temperature (25 °C) until cooling to 40 °C. The conditions predicted by the models successfully yielded creams that met all specified targets. Creams compounded under optimal conditions exhibited well-defined oil droplets, with uniform droplet size in aqueous cream and mild size heterogeneity in cetomacrogol cream. Freeze-thaw testing demonstrated that both optimised creams were stable with no observable phase separation. **Conclusions**: This study demonstrates that a systematic experimental approach to optimising compounding parameters for the APF aqueous cream and cetomacrogol cream resulted in high-quality, stable, and reproducible products. Formulary guidelines, such as the APF, could benefit from adopting QbD approaches to improve the standardisation of compounding instructions in pharmacy practice.

## 1. Introduction

Quality by Design (QbD) is a systematic, risk-based, and regulatory-endorsed framework that embeds quality and reliability into a medicinal product from the earliest design stages, thereby ensuring that products released to patients are consistently of high quality. The QbD approach involves defining the critical quality attributes (CQAs) of the medicine, which are the measurable parameters that determine its safety, quality and efficacy, and understanding how critical process parameters (CPPs) in the manufacturing process affect these CQAs. A Design of Experiments (DoE) strategy provides the structured experimentation and statistical power needed to elucidate these relationships, offering clear advantages over traditional one-factor-at-a-time studies by capturing factor interactions and supporting final decisions with robust statistics [1].

Despite its growing role in industrial pharmaceutical product development, QbD has rarely been applied to medicines compounded in community or hospital pharmacies. Compounding is required when a suitable commercial product is unavailable [1,2]. In many jurisdictions, including the USA, the UK and Australia, regulatory agencies do not provide batch-by-batch oversight of compounded medicines, and their quality, safety and efficacy are the responsibility of the compounding pharmacist. Although compounding pharmacists must abide by good compounding practices guidelines set up by professional governing bodies, they generally do not develop formulations de novo, instead relying on pharmacopoeias, formularies, and professional compounding associations for instructions. Given this reliance, incorporating a QbD philosophy during the preparation of these professional guidelines could give pharmacists deeper insight into CPPs and greater capacity to mitigate risks and provide products that consistently meet the specific patient needs.

This study explores the feasibility of employing a QbD approach for compounded medicines, using two simple water-based creams as model products. Creams are complex semi-solid emulsions where an oil phase is emulsified into an aqueous phase [3]. If poorly prepared, they may undergo coalescence, crystallisation, or creaming, resulting in phase separation and reduced efficacy [4,5]. They must also exhibit acceptable rheology, presenting as an aesthetically pleasing thick cream in the container yet spreading easily on skin [6,7]. The stability of creams is largely determined by the ingredients used [8,9,10], and the effect of different ingredients and how they can be manipulated to optimise the cream formulation has been well-studied [11,12,13,14,15,16,17,18]. An example is a study by Alves et al., who successfully employed the DoE approach to optimise a clotrimazole cream formulation through the selection of ingredients and their quantities [1]. The influence of the processing conditions employed in the compounding of creams has, however, been largely neglected [19]. The combined effect of multiple processing parameters, as well as the optimal conditions for preparation, is yet to be quantified for creams compounded in a pharmacy setting [19,20].

We therefore applied a factorial DoE to identify optimal processing conditions for two formulations listed in the Australian Pharmaceutical Formulary (APF), Aqueous Cream and Cetomacrogol Cream Aqueous (Table 1). These creams have appeared in successive APF editions since 1921 [7,21] and are widely compounded across Australia. These creams are primarily used as emollient bases for moisturising and soothing dry skin conditions, and also serve as versatile vehicles for the incorporation of various active pharmaceutical ingredients (APIs) to treat a range of dermatological conditions. Yet even the latest APF instructions contain ambiguities such as “combine the two phases and stir,” without specifying temperature, speed, or duration [7]. In medicine compounding laboratory classes conducted at The University of Western Australia, 10–30% of pharmacy students fail to produce an acceptable Aqueous Cream when following the current APF method. Accordingly, we aimed to specify optimised compounding steps using the DoE approach. Firstly, CPPs embedded in the APF procedure were identified: oil phase temperature, water phase temperature, mixing-environment temperature, stirring speed, and endpoint temperature during phase combination [7]. These factors were varied systematically, while the CQAs—physical appearance, stability, spreadability, creaming propensity, and viscosity [22]—served as response variables. We hypothesised that one or more CPPs would significantly influence the CQAs and that an optimal parameter combination could be defined for each cream.

## 2. Materials and Methods

### 2.1. Materials

For preparing the aqueous creams, emulsifying ointment BP was purchased from PharmAust (Perth, Australia), glycerol analytical reagent from ChemSupply (Adelaide, Australia), and phenoxyethanol USP from Sigma Aldrich (Melbourne, Australia). For preparing cetomacrogol creams, cetomacrogol emulsifying wax BP was purchased from PharmaScope (Perth, Australia), propylene glycol BP, liquid paraffin BP and white soft paraffin BP from PharmAust (Perth, Australia), and chlorocresol from Sigma Aldrich (Melbourne, Australia). Water for both creams was from a reverse osmosis system (PSI Waters Australia, Model: BOSS 031-4P, Launceston, TAS, Australia). Kenkay Dry Skin Aqueous Cream BP© and David Craig Sorbolene Cream© purchased from local pharmacies (Perth, Australia) served as the respective reference creams for the aqueous creams and cetomacrogol creams prepared in this study.

### 2.2. Methods

#### 2.2.1. Experimental Design

Response Surface Model (RSM) was employed, and experiments were designed using a 3-level factorial design to determine the effects of five processing conditions on the cream quality. Independent variables were the temperature of the oil phase (A), temperature of the water phase (B), and the temperature of the stirring environment (C, a proxy for the cooling rate of the combined phases, with lower values indicating more rapid cooling), stirring speed (D), and temperature at which stirring was ceased (E, final temperature). Cream quality was evaluated through three dependent variables. These were spreadability, viscosity and creaming index. Each factor was evaluated at three levels: the minimum (−1), midpoint (0), and maximum (+1). Preliminary experiments were conducted to determine the −1 and +1 levels, with the level 0 value defined as the midpoint between these extremes (Table 2). The Stat-Ease 360© (version 22.0, Minneapolis, MN, USA) was used to generate 32 randomised combinations of processing conditions. Design points were selected based on a Central Composite Design (CCD) and consisted of 26 non-centre points and 6 centre points, to give a final total of 32 samples to prepare various aqueous and cetomacrogol creams (Table S1).

#### 2.2.2. Cream Preparation

The 32 aqueous and cetomacrogol cream samples were compounded using the formulas in the APF 26th edition (Table 1) [7]. Each ingredient was measured by weight, and cream samples were prepared to a final weight of 100 g (Table 3).

Separate oil phases and water phases were prepared for each cream, using the ingredients specified in the APF (Table 1). Chlorocresol a 0.4% stock solution was used for the compounding of the 32 DoE creams as well as the optimal cetomacrogol creams [23,24] The stock solution was prepared by dissolving 4 g of chlorocresol in 250 mL of freshly boiled water (heated to 100 °C), followed by sonicating (Unisonics^®^ Sonicator, SN2112, Brookvale, NSW, Australia) for an hour and adjusting to a volume of 1 L with water after cooling to 25 °C. The chlorocresol stock solution was stored at ambient temperature and utilised within seven days. To compound 100 g of cetomacrogol cream, a 25 mL aliquot of the stock solution was added to the water phase to incorporate 100 mg of chlorocresol (Table 3).

The oil phase and water phases were heated to the specified temperatures (Table 2) using a water bath set at 95 °C (BUCHI Labortechnik AG^®^, SN1000020861, Braeside, VIC, Australia). For experiments where the water phase was required at the +1 level temperature (>80 °C), the water phase was first heated to 80 °C on the water bath, and a hotplate (Industrial Equipment & Control^®,^, Thornbury, VIC, Australia) was used to further bring the water phase to the final temperature. The temperatures of the oil and water phases during heating were monitored using thermometers. Once the target temperature was reached, the oil phase was immediately transferred to a ceramic evaporating dish placed in a water bath set atop a Radley© magnetic stirrer (SN200081056, Saffron Walden, UK). The water phase was added to the oil phase, and a 5 cm magnetic stirring rod was used for mixing the two phases at the specified revolutions per minute (rpm). The water bath consisted of a plastic container (9.5 × 13 × 13 cm) filled to a quarter with water and containing a thermometer. The bath temperature was maintained at the specified temperature (±1 °C) using warm water or ice. A thermometer was also placed in the evaporating dish, and stirring of the two phases was ceased once the resultant cream had reached its designated end temperature (Table 2).

To enable the high number of samples to be prepared and evaluated immediately after compounding, the 32 DoE cream samples were compounded across two days, with a one-day interval inserted between the two manufacturing days to allow for the creams prepared on the previous day to be evaluated. This evaluation provided the baseline characteristics for the cream samples. Cream samples were stored at 25 °C in 100 g opaque plastic jars until analysis.

#### 2.2.3. Evaluation of Sample Creams

##### Physical Appearance

The visual assessment was conducted under standardised laboratory lighting conditions. Each cream sample was visually assessed for colour, texture, odour and phase separation, which was deemed to have occurred if visible oil drops or layers were observed on the cream surface.

##### Spreadability

**Figure 1:**
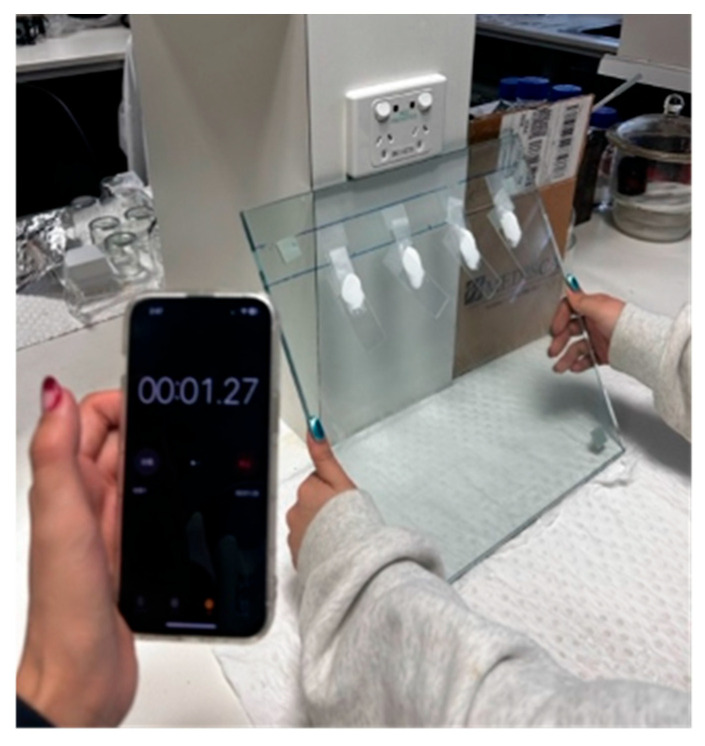
Set-up of spreadability test. Commercial cream is on the far left. Stop-watch was used to record the time taken for each slide to fall.

Spreadability of cream samples was tested at 25 °C using the parallel plate method (Figure 1) described by Jelvehgari et al. with modifications [25]. Guided by a template, a 300-mg aliquot of cream sample was dispensed centrally onto a microscope glass slide (Livingstone, Mascot, NSW, Australia) using a 1 mL oral syringe (with the tip cut off). The glass slides were placed evenly apart on a larger glass slab (34 × 34 cm, thickness 0.8 cm) and secured in position with sticky tape. A second glass slide was placed on top of each glass slide to compress the cream sample, and after one minute, the glass slab was lifted to an angle of 53° relative to a flat benchtop (Figure 1). Timing commenced as soon as the slab was lifted, and the time taken for the free glass slide to slip past the fixed slide was noted. Each glass slab contained a triplicate of a sample cream and a control cream sample comprising the respective commercial cream, with new microscope slides used for all experiments. The spreadability ratio was calculated as the time (in seconds) taken for each sample’s slide to fall relative to the time taken for the respective control (commercial cream) sample’s slide to fall (Equation (1)). Cream samples that did not show slide slippage after 3 min were assigned a time of 180 s for DoE analysis purposes.

(1) $$\mathrm{Spreadability}\;\mathrm{Ratio}\;=\frac{\mathrm{Time}\;\mathrm{taken}\;\mathrm{for}\;\mathrm{test}\;\mathrm{sample}\;\mathrm{slide}\;\mathrm{to}\;\mathrm{fall}\;(\mathrm{seconds})}{\mathrm{Time}\;\mathrm{taken}\;\mathrm{for}\;\mathrm{commercial}\;\mathrm{sample}\;\mathrm{slide}\;\mathrm{to}\;\mathrm{fall}\;(\mathrm{seconds})}$$

##### Viscosity

Dynamic viscosity measurements of the cream samples were conducted under ambient conditions using a viscometer (John Morris ATAGO digital, SN6800-E07, Bentley, Western Australia, Australia) equipped with an A3 spindle set to rotate at 20 rpm for 15 mL aqueous cream samples, and 10 rpm for the 15 mL cetomacrogol cream samples. Triplicate measurements were taken for each sample, with a 1 min recovery time between measurements. Unless otherwise specified, the term ‘viscosity’ in this manuscript refers to dynamic viscosity.

##### Creaming Index

**Figure 2:**
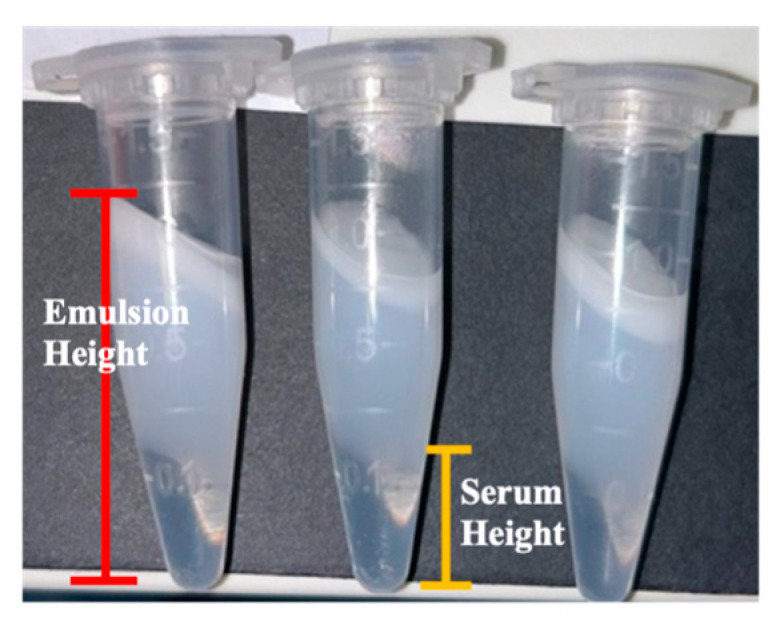
Triplicates of cream samples after centrifugation at a speed of 13,400 rpm for 30 min.

Cream samples were assessed for their propensity to creaming using a centrifugation method. Triplicate 1 g samples were centrifuged at 13,400 rpm for 30 min (Eppendorf MiniSpin© Centrifuge, SNT12W-883072, Macquarie Park, NSW, Australia) and the height of the clear serum layer was measured relative to the total height of the cream emulsion (Equation (2), Figure 2) using a 30 cm ruler. The creaming index was calculated using the following Equation (2) [26]:

(2) $$\mathrm{Creaming}\;\mathrm{Index}\;(\mathrm{CI})\;\%=\frac{\mathrm{Height}\;\mathrm{of}\;\mathrm{serum}\;\mathrm{layer}\;(\mathrm{cm})\;}{\mathrm{Total}\;\mathrm{height}\;\mathrm{of}\;\mathrm{the}\;\mathrm{emulsion}\;(\mathrm{cm})}\;\times \;100\%$$

#### 2.2.4. Optimisation of Cream Compounding

The spreadability, viscosity and creaming responses measured for the 32 samples of aqueous cream and cetomacrogol cream were analysed using Stat-Ease 360© software, with statistical significance defined at *p* < 0.05. Optimal processing conditions were then predicted using appropriate mathematical models.

Target responses for the optimal creams were set at a spreadability ratio of 1 (spreadability comparable to commercial control) and creaming index of 0% (no phase separation). The target viscosity for the aqueous cream was able to be set to match that of the commercial cream, whereas the target viscosity for the cetomacrogol cream was set at 150% of the commercial cream viscosity. This was because the commercial cetomacrogol cream had lower viscosity than all except for one cetomacrogol DoE samples, and it was also not more stable than the test samples. Additionally, the cetomacrogol cream samples with viscosity at 150% of the commercial cream exhibited acceptable physical appearance.

#### 2.2.5. Confirmation Studies of Optimal Aqueous and Cetomacrogol Creams

##### Preparation and Evaluation of Optimal Creams

Triplicate batches of the optimised aqueous cream and cetomacrogol cream were prepared according to the model-predicted conditions, following the procedures described in Section 3.2. On the day following preparation, each optimised cream was evaluated for physical appearance, spreadability, viscosity, and creaming index, using methods as described in Section 3.3.

##### Freeze-Thaw Cycle

The optimised creams and their respective commercial controls were further evaluated for stability using the freeze-thaw cycle technique. Each sample was subject to five freeze-thaw cycles over ten days, simulating temperature variations in real life that occur during storage in different climate zones [27]. Samples were frozen for 24 h at −20 °C and thawed for 24 h at ambient conditions in 10 mL clear glass vials, filled to a height of 2.8 cm. Following visual inspection for changes in physical appearance and phase separation, the thawed samples were returned to the freezer to commence the next freeze-thaw cycle. If no visible phase separation was observed, the cream was considered stable.

##### Optical Microscopy

The microstructure, in particular the homogeneity of oil droplet size, of the optimised aqueous and cetomacrogol creams, along with their respective commercial controls, was observed at 20X magnification using an Olympus CKX41 microscope (Sanford, NC, USA).

##### Data Analysis

The spreadability, viscosity, and creaming index of the optimised aqueous and cetomacrogol creams were evaluated to confirm that the identified optimal processing conditions reliably predicted these target quality attributes. Statistical analysis was conducted using Stat-Ease^®^ 360 software, confirming that the mean values for spreadability, viscosity, and creaming index of the triplicate optimised creams fell within the 95% confidence intervals predicted by the respective models.

## 3. Results

### 3.1. DoE Cream Samples

#### 3.1.1. Physical Appearance

The 32 aqueous cream samples differed significantly in appearance. Most samples (*n* = 24) exhibited some level of glossiness, with 6 samples assessed to be highly glossy and smooth in texture. Eight samples had a thick oily cap above a watery phase, indicating phase separation.

Thirty of the 32 cetomacrogol cream samples exhibited glossiness, with 5 samples assessed to be highly glossy and smooth in texture. All samples had a mild odour attributable to chlorocresol. All 32 samples were thick creams, and none displayed phase separation.

#### 3.1.2. Spreadability

The aqueous cream samples exhibited wide variation in spreadability, ranging from 0 to 35 s. A linear model provided the best fit for the data, with the lack-of-fit test showing no significance (*p* = 0.5397), indicating a good overall model fit (Table 4). Among the processing variables, stirring speed was identified as the only significant factor influencing the spreadability of the aqueous creams. Specifically, higher stirring speeds were associated with increased cream spreadability, as reflected by a positive model coefficient. Poor cream spreadability (spreadability ratio ≤ 0.2) was observed using the −1 level stirring speed condition (100 rpm), likely due to insufficient emulsification of the oil phase into the aqueous phase. These poorly spreadable aqueous cream samples exhibited a thin, watery consistency.

The predicted model for cetomacrogol cream spreadability was a two-factor interaction model with no significant lack of fit (Table 5), indicating a good overall fit. While no individual processing parameter had a dominant effect on cream spreadability, which ranged from 11 to 180 s, significant interactions between factors were identified. Specifically, the interaction between water phase temperature and stirring speed, as well as the interaction between stirring environment temperature and the final temperature at which stirring was stopped, significantly influenced cream spreadability. Compared to the commercial cetomacrogol cream sample, several test samples (*n* = 5) demonstrated reduced spreadability, with two samples requiring more than three minutes to spread.

#### 3.1.3. Viscosity

The modified quadratic model provided the best fit for the viscosity data, which ranged from 286 mPa·s to 35,086 mPa·s for the aqueous cream samples, compared to the control (13,751 ± 1785 mPa·s, *n* = 3), despite a significant lack of fit. Cream viscosity was significantly influenced by the water phase temperature, stirring speed, and stirring environment temperature. Slower cooling, corresponding to higher temperatures, tended to increase final cream viscosity. Conversely, higher stirring rates reduced the cream viscosity, as indicated by the large negative coefficient of the quadratic term for stirring speed (D^2^ in Table 6).

For the cetomacrogol cream samples, the linear model provided the best fit for the viscosity data, with no significant lack of fit (Table 7). The only significant factor influencing cream viscosity was the temperature of the water phase, with higher water phase temperatures increasing cream viscosity. Despite wide variability in viscosity among the 32 cetomacrogol cream samples (29,087 ± 3461 mPa·s to 103,341 ± 752 mPa·s), most creams (*n* = 31) recorded viscosities exceeding that of the commercial cream (37,376 ± 7266 mPa·s). The majority of the cetomacrogol creams exhibited a thick consistency, such that when stirred, they retained the marks or indentations from stirring and did not flow back or return to a smooth, uniform surface, indicating the high thickness and rigidity of the cream.

#### 3.1.4. Creaming Index

The creaming index for the 32 aqueous cream samples ranged from 0% to 95.6%, while the commercial cream had an index of 22.2% ± 1.8%. For the creaming index response, the linear model fitted well with no lack of fit, and the temperature of the water phase and stirring speed were identified as significant factors (Table 8). Higher water phase temperature and stirring speed reduced the creaming index, indicating that these processing conditions positively impacted the aqueous cream stability by reducing creaming propensity.

The reduced quadratic model provided the best fit for the creaming index responses of the cetomacrogol cream samples, with no lack of fit (Table 9). The temperature of the water phase and stirring speed were identified as significant factors, and the two factors also had a significant interaction. The negative and larger absolute value of the coefficient for the quadratic term (D^2^) of stirring speed suggests that higher stirring speeds might reduce creaming in a non-linear pattern. In general, the majority of cetomacrogol cream samples (*n* = 24) displayed no creaming, indicating that cetomacrogol cream was more stable and less influenced by process conditions compared to aqueous cream. The cetomacrogol cream samples had a narrower creaming index compared to the aqueous cream samples, with samples showing the greatest phase separation having a creaming index of 48%. The other cetomacrogol cream samples had a low creaming index (≤10%) relative to the commercial sample, which exhibited a creaming index of 29.5% ± 2.2%, indicating that it was less stable than the compounded samples.

### 3.2. Determination of Optimal Processing Conditions

For each cream formulation, the three responses were analysed, each fitted to its best model. The profile plots, consisting of 20 panels, each depicting the effect of one factor on a specific response, clearly highlight that stirring speed (D) was the most influential processing condition affecting all measured responses of the aqueous cream samples (Figure S1A). Conversely, the measured responses of the cetomacrogol cream samples were less affected by the processing conditions applied, with the temperature of the water phase (B) shown to be the most influential processing condition on the viscosity of the cetomacrogol cream (Figure S1B).

The optimal final process conditions required to achieve the targets for all three responses in each cream formulation were determined using a desirability function, calculated as the geometric mean of the individual rankings for achieving each target response, as outlined in Section 2.2.4.

Conditions achieving a desirability value above 0.7 were considered optimal for this study. The maximum desirability value for the aqueous cream samples to meet all targets was 0.73; the range of optimum conditions is summarised in Table 10. The optimum oil phase temperature was lower than the water phase temperature, and the mixing environment for the two phases needed to be cold. Interestingly, ceasing stirring of the two phases at 50 °C yielded the optimal results. Within this range, the conditions selected for the confirmation study were: oil phase temperature of 60 °C, water phase temperature of 80 °C, phase mixing to be conducted using an ice bath (10 °C) at a stirring speed of 250 rpm and stopping the mixing at a cream temperature of 50 °C.

For the cetomacrogol cream, a desirability value of 1 was targeted, and the optimal conditions are summarised in Table 10. A range of oil phase temperatures (60–80 °C) can be used, and the final temperature can be flexible (ambient to 50 °C); however, the water phase temperature must be set at 75 °C. Within the optimal range, the conditions selected for the confirmation study were: oil phase temperature of 70 °C, water phase temperature of 75 °C, phase mixing to be conducted at ambient temperature (25 °C) at a stirring speed of 220 rpm and stopping the stirring at 40 °C.

### 3.3. Confirmation Studies of Optimised Creams

#### 3.3.1. Evaluation of Optimised Creams

Immediately after preparation, following the established optimal conditions, the optimised aqueous cream (*n* = 3) was slightly runny; however, once left to cool to ambient temperature after stirring ceased at 50 °C, the samples attained a semi-solid consistency. All three optimal aqueous creams had a white, glossy appearance with smooth texture and no discernible phase separation. They had similar spreadability but were more viscous (23,000 to 27,000 mPa·s) compared to the commercial cream. All three optimal aqueous creams exhibited a creaming index of 0% while the commercial cream recorded a creaming index of 17.2% ± 1.9%. Confirmational analysis showed the spreadability and viscosity responses to be within the prediction interval, confirming that the model accurately predicted the optimal conditions to meet the desired targets for the aqueous cream.

All three optimised cetomacrogol creams had a white, glossy appearance with smooth texture and no discernible phase separation. The optimised creams exhibited 20% higher spreadability relative to the commercial cream and had a creaming index of 0% whereas the commercial cream had a creaming index of 29.5% ± 2.2%. The viscosity for the optimised creams ranged from 64,000 to 68,000 mPa·s, which was within the predicted range. Of note, the measured responses for the three optimal creams had standard deviations of ± 0%, indicating high levels of replicability. While the creaming index responses could not be fitted to a confidence interval as the standard deviation was ± 0%, the viscosity and spreadability responses for the optimal cetomacrogol creams were confirmed to be within the 95% prediction intervals of model prediction.

#### 3.3.2. Freeze-Thaw Cycle

**Figure 3:**
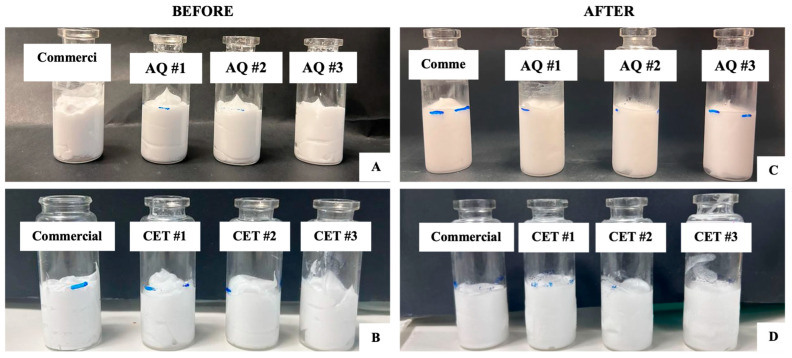
Physical appearance of the optimal creams before and after the ten-day freeze-thaw testing. (**A**): Optimal aqueous creams before. (**B**): Optimal cetomacrogol creams before. (**C**): Optimal aqueous creams after freeze-thaw testing were complete. (**D**): Optimal cetomacrogol creams after freeze-thaw testing were complete.

The optimised aqueous creams and cetomacrogol creams were subjected to five cycles of freeze-thaw. None of the samples exhibited any apparent changes in physical appearance following each cycle of testing (Figure 3). Neither did any of the samples exhibit discernible phase separation.

#### 3.3.3. Optical Microscopy

**Figure 4:**
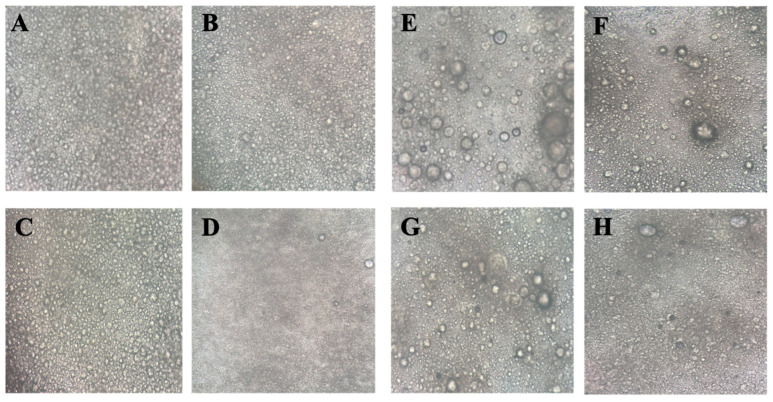
Microstructure of the optimal creams. Images observed at 20X magnification under an optical microscope (Olympus CKX41). (**A**). Aqueous optimal cream #1. (**B**). Aqueous optimal cream #2. (**C**). Aqueous optimal cream #3. (**D**). Commercial Aqueous cream BP. (**E**). Cetomacrogol optimal cream #1. (**F**). Cetomacrogol optimal cream #2. (**G**). Cetomacrogol optimal cream #3. (**H**). Commercial cetomacrogol cream. The images are for qualitative assessment only, and no quantitative analysis of droplet size should be inferred due to the absence of a scale.

The optimised aqueous creams, when viewed under the optical microscope, displayed a uniform distribution of fine droplets that were relatively homogeneous in size (Figure 4). Their appearance was similar to each other and to the commercial cream. The optimised cetomacrogol creams, when viewed under the microscope, had a less uniform distribution of droplets that varied more widely in size distribution. However, their appearance was similar to that of the commercial cream and to each other. In both cream formulations, the droplets in the cream samples were not as densely packed as in their commercial counterparts, possibly because the commercial creams had been subjected to homogenisation to reduce their droplet size [28].

## 4. Discussion

This study has demonstrated the feasibility of applying the QbD approach to optimising the compounding of two aqueous cream formulations. Using the DoE model, we systematically determined the optimal processing conditions for compounding the APF aqueous cream and cetomacrogol cream. The DoE approach to optimisation produces more cost-effective and reliable results than the traditional OFAT approach, and is the preferred approach endorsed by regulatory authorities for designing studies aimed at optimising medicinal formulations [29]. The DoE model had previously been applied to produce a replicable cream formulation of high quality [30]. This utility was also demonstrated by Alves et al., who utilised a QbD approach to develop an optimised clotrimazole cream [1]. In the DoE model, the experimental trials alongside past experience and literature reviews are essential in defining the experimental design space. If the design space is not appropriately established, the resulting optimum conditions may be misleading. In our study, the experimental design was well within the desired space, and the optimum cream demonstrated good consistency, as demonstrated by the results of the confirmation study. Thus, the feasibility of using the QbD approach to optimise the compounding of the APF aqueous cream and cetomacrogol cream.

The definition of the optimal compounding conditions for the APF aqueous cream and cetomacrogol cream is a novel finding that is yet unexplored by other studies in the literature. While there have been countless studies investigating the optimisation of cream formulations by focusing on identifying optimal ingredients and their quantities to develop new cream formulations [11,12,13,14,15,16,17,18], there have been relatively few studies seeking to optimise the compounding of products listed in existing formularies. While many compounding pharmacies rely on commercially available cream bases for API incorporation, the extemporaneous compounding of aqueous and cetomacrogol creams continues to be part of the practice skills taught in pharmacy schools. Understanding the processing variables that most significantly influence the quality of these creams provides valuable insights that may inform compounding practices more broadly. Our study fills this gap in the literature.

This study demonstrated that among the identified CPPs, stirring speed during the mixing of the oil and water phases was the most influential processing parameter affecting all three quality attributes of the APF aqueous cream. Notably, high stirring speeds facilitated the formation of a stable primary emulsion even when stirring was stopped at 50 °C; although stirring was discontinued at this temperature, the cream remained stable and did not break down. If the emulsion remained relatively thin at this point, brief additional stirring was sufficient to produce a stable, well-formed cream. In contrast, the quality of the APF cetomacrogol cream was less influenced by processing conditions, although the temperature of the water phase had a relatively significant effect on cream viscosity. These findings suggest that cetomacrogol cream is less sensitive to the tested processing factors compared to aqueous cream, indicating greater formulation robustness. Furthermore, these results highlight that the optimised compounding conditions cannot be generalised but must be individually determined for each cream formulation. Our findings also do not contradict the findings of the limited published literature on cream compounding. Realdon et al. in a 2001 study [20] concluded that using different stirring equipment, in their case a turbo-mixer and a hand blender, for preparing creams could result in products of different viscosity and droplet size. Cunningham et al. also found that mixing speed and temperature significantly impact the viscosity of prepared semi-solids [19].

Over the years, the APF instructions for compounding aqueous and cetomacrogol creams have been updated multiple times [31,32,33,34,35,36,37]. Yet, certain ambiguities persist, particularly regarding aqueous cream. Because reproducible processes are essential for ensuring consistent cream quality, practical measures for standardising compounding procedures are needed. Based on the optimal conditions identified in this study, we propose the following revised instruction: Aqueous Cream—‘Add glycerol and phenoxyethanol to 60 mL of purified water and heat the mixture to approximately 80 °C. Separately, melt the emulsifying ointment at 60 °C. Combine the two phases and stir vigorously (250 rpm if a calibrated stirrer is available) until a semi-solid cream forms and the temperature is 50 °C. Emphasising ‘stir vigorously’ is essential because stirring speed was found to be critical for achieving the target cream quality.

Cetomacrogol cream demonstrated relatively high robustness, indicating that its compounding instructions may not need to be as stringent as those for aqueous cream. However, maintaining the specified water phase temperature of 75 °C remains important for ensuring cream quality and should be explicitly included in the compounding procedure.

By incorporating these more precise processing conditions—particularly with respect to temperature and stirring speed—the compounding of both aqueous and cetomacrogol creams can be made more consistent and reproducible. Even minor refinements to standard procedures can significantly enhance the likelihood of consistently achieving the desired cream characteristics in routine compounding practice.

Despite the strengths of this study, several limitations should be acknowledged. One limitation was the use of only a single commercial product as the control for each cream formulation. Other commercial aqueous and cetomacrogol creams, produced under different manufacturing conditions, may exhibit varying properties compared to the two commercial reference creams used in this study. Incorporating the average properties of multiple commercial creams to define target CQAs may offer a more representative benchmark and enhance the generalisability of the findings. Another limitation was the spreadability evaluation, although its inherent variability across runs [17] was mitigated by normalising the results using the commercial control that was included in the same run. Measurement of shear using a texture analyser may provide more objective comparisons of spreadability between the formulations. Another alternative is the spreadability test described by Contreras and Sanchez [15], which involves using weights to press a cream sample onto a glass slab and measuring the diameter of the spread. Lastly, while this study has defined the optimal processing conditions for compounding the APF aqueous and cetomacrogol creams, further studies that incorporate quantitative analyses of viscosity, pH, and droplet size distribution following the accelerated stability testing will be required to complement the visual assessments and further strengthen the understanding of cream stability.

## 5. Conclusions

This study has highlighted the feasibility and advantages of applying QbD principles and a DoE approach to optimise the compounding of the APF aqueous cream and cetomacrogol cream. By systematically identifying and controlling critical process parameters, particularly temperature and stirring speed, both formulations can be prepared with greater consistency and reproducibility. In particular, the quality of the APF aqueous cream was found to be highly sensitive to the stirring speed employed during manufacture, whereas the cetomacrogol cream formulation was more robust but still benefited from attention to the temperature of the aqueous phase during preparation. Our proposed compounding instructions address these factors, offering practical means to improve cream quality in pharmacy settings. Although this study focused only on two specific commercial products and employed particular evaluation methods (e.g., spreadability testing), the findings underscore the importance of precisely defined compounding procedures.

## Figures and Tables

**Table 1:** Formulations and instructions provided in the Australian Pharmaceutical Formulary (APF), 26th edition, for the compounding of Aqueous Cream and Cetomacrogol Cream Aqueous.

	APF Aqueous Cream	APF Cetomacrogol Cream Aqueous
**Formulation**	Emulsifying ointment 30 g Glycerol 5 mL Phenoxyethanol 1 g Purified water, freshly boiled and cooled, to 100 g	Cetomacrogol emulsifying wax 15 g Liquid paraffin 10 g White soft paraffin 10 g Chlorocresol 100 mg Propylene glycol 5 mL Purified water, freshly boiled and cooled, to 100 g
**Compounding Instructions**	Add glycerol and phenoxyethanol to 60 mL of purified water and heat to approximately 60 °C. Separately melt the emulsifying ointment. Combine the two phases and stir until a semi-solid cream forms. Make up to 100 g with warm purified water and mix thoroughly. Stir until cool.	Melt the cetomacrogol emulsifying wax in the paraffins at about 70 °C. Add the chlorocresol to a warmed 200 mL container, then add 60 mL of just-boiled purified water (>80 °C), close the container and shake to dissolve. Immediately add the propylene glycol to the aqueous phase, then mix both phases and stir until a semi-solid cream forms. Make up to 100 g with warm purified water and mix thoroughly. Stir until cool.

**Table 2:** Values assigned to processing conditions A, B, C, D and E at the levels −1, 0, and +1 for preparing aqueous and cetomacrogol cream samples, for the Design of Experiments.

Processing Condition	Unit of Measure	Aqueous Cream Levels			Cetomacrogol Cream Levels		
		−1	0	+1	−1	0	+1
A ^1^	°C	60	70	80	60	70	80
B ^2^	°C	60	75	90	75	85	95
C ^3^	°C	30	20	10	30	20	10
D ^4^	rpm	100	200	300	100	200	300
E ^5^	°C	30	40	50	30	40	50

^1^ Temperature of the oil phase, ^2^ Temperature of the water phase, ^3^ Temperature of the stirring environment, which indicates the rate of cooling (lower values signify a faster cooling rate), ^4^ Stirring speed, ^5^ Final temperature at which stirring was ceased.

**Table 3:** Quantities of ingredients used to compound APF aqueous and cetomacrogol creams. Formulae were adapted from APF 26th edition [3]. Creams were made to a total weight of 100 g. All quantities are expressed by weight.

Formulation	Phases	Ingredients	Quantities (g)
APF Aqueous Cream	Water	Glycerol	6.3
		Phenoxyethanol	1
		Water	62.7
	Oil	Emulsifying Ointment BP	30
TOTAL			100
APF Cetomacrogol Cream	Water	Chlorocresol	0.1
		Propylene Glycol	5.2
		Water	34.8
	Oil	Cetomacrogol Emulsifying Wax	15
		Liquid Paraffin	10
		White Soft Paraffin	10
TOTAL			100

**Table 4:** Analysis of variance (ANOVA) for the effect of stirring speed (D) on the normalised square root of spreadability of aqueous cream and coefficient of the final equation in terms of coded factors.

Source	Sum of Squares	df	Mean Square	F-Value	*p*-Value	Coefficient of Final Equation in Terms of Coded Factors
Model	2.22	1	2.22	32.63	<0.0001	
D	2.22	1	2.22	32.63	<0.0001	0.351
Residual	2.04	30	0.068			
Lack of Fit	1.71	25	0.0684	1.04	0.5397	
Pure Error	0.3286	5	0.0657			
Cor Total	4.26	31				

**Table 5:** Analysis of Variance (ANOVA) for the effect of processing conditions and their interactions on the normalised spreadability of cetomacrogol cream and coefficient of the final equation in terms of coded factors. B: Temperature of water phase, C: Temperature of stirring environment, D: Stirring speed, E: End temperature of stirring.

Source	Sum of Squares	df	Mean Square	F-Value	*p*-Value	Coefficient of Final Equation in Terms of Coded Factors
Model	3.29	7	0.4702	3.27	0.0138	
B	0.2689	1	0.2689	1.87	0.184	0.1222
C	0.245	1	0.245	1.71	0.204	0.1167
D	0.0022	1	0.0022	0.0155	0.9021	0.0111
E	0.08	1	0.08	0.5568	0.4628	0.0667
BD	0.81	1	0.81	5.64	0.0259	−0.225
CE	1.32	1	1.32	9.2	0.0057	0.2875
DE	0.5625	1	0.5625	3.91	0.0595	0.1875
Residual	3.45	24	0.1437			
Lack of Fit	2.59	19	0.1362	0.7921	0.6797	
Pure Error	0.86	5	0.172			
Cor Total	6.74	31				

**Table 6:** Analysis of Variance (ANOVA) for the effect of processing conditions and their interactions on the viscosity of aqueous cream and coefficient of the final equation in terms of coded factors. A: Temperature of oil phase, B: Temperature of water phase, C: Temperature of stirring environment, D: Stirring speed, E: End temperature of stirring.

Source	Sum of Squares	df	Mean Square	F-Value	*p*-Value	Coefficient of Final Equation in Terms of Coded Factors
Model	2.82 × 10^9^	11	2.56 × 10^8^	39.94	<0.0001	
A	6.58 × 10^6^	1	6.58 × 10^6^	1.03	0.3231	604.5
B	8.05 × 10^7^	1	8.05 × 10^7^	12.56	0.002	2115.06
C	4.21 × 10^7^	1	4.21 × 10^7^	6.56	0.0186	1528.61
D	1.77 × 10^9^	1	1.77 × 10^9^	276.63	<0.0001	9924.5
E	12,534.72	1	12,534.72	0.002	0.9652	26.39
AD	3.44 × 10^7^	1	3.44 × 10^7^	5.37	0.0312	1466.25
AE	3.03 × 10^7^	1	3.03 × 10^7^	4.72	0.0419	1375.5
BD	2.57 × 10^7^	1	2.57 × 10^7^	4.01	0.0589	1267.62
BE	2.76 × 10^7^	1	2.76 × 10^7^	4.3	0.0512	−1312.37
CE	4.67 × 10^7^	1	4.67 × 10^7^	7.28	0.0138	1708
D^2^	7.49 × 10^8^	1	7.49 × 10^8^	116.85	<0.0001	−9751.73
Residual	1.28 × 10^8^	20	6.41 × 10^6^			
Lack of Fit	1.22 × 10^8^	15	8.13 × 10^6^	6.53	0.0242	
Pure Error	6.23 × 10^6^	5	1.25 × 10^6^			
Cor Total	2.94 × 10^9^	31				

**Table 7:** Analysis of Variance (ANOVA) for the effect of water phase temperature (B) on the viscosity of cetomacrogol cream and coefficient of the final equation in terms of coded factors.

Source	Sum of Squares	df	Mean Square	F-Value	*p*-Value	Coefficient of Final Equation in Terms of Coded Factors
Model	5.35 × 10^9^	1	5.35 × 10^9^	33.06	<0.0001	
B	5.35 × 10^9^	1	5.35 × 10^9^	33.06	<0.0001	17,239.67
Residual	4.86 × 10^9^	30	1.62 × 10^8^			
Lack of Fit	4.48 × 10^9^	25	1.79 × 10^8^	2.39	0.1687	
Pure Error	3.75 × 10^8^	5	7.50 × 10^8^			
Cor Total	1.02 × 10^10^	31				

**Table 8:** Analysis of Variance (ANOVA) for the effect of water phase temperature (B) and stirring speed (D) on the normalised square root of creaming of aqueous cream and coefficient of the final equation in terms of coded Factors.

Source	Sum of Squares	df	Mean Square	F-Value	*p*-Value	Coefficient of Final Equation in Terms of Coded Factors
Model	196.75	2	98.38	17.03	<0.0001	
B	35.14	1	35.14	6.08	0.0198	−1.4
D	161.61	1	161.61	27.98	<0.0001	−3
Residual	167.51	29	5.78			
Lack of Fit	140.42	24	5.85	1.08	0.5187	
Pure Error	27.1	5	5.42			
Cor Total	364.27	31				

**Table 9:** Analysis of Variance (ANOVA) for the effect of water phase temperature (B), stirring speed (D) and their interactions on 1/Sqrt (Creaming + 0.10) of cetomacrogol cream and coefficient of the final equation in terms of coded Factors.

Source	Sum of Squares	df	Mean Square	F-Value	*p*-Value	Coefficient of Final Equation in Terms of Coded Factors
Model	27.36	5	5.47	6.6	0.0004	
B	10.71	1	10.71	12.91	0.0013	0.7713
D	3.38	1	3.38	4.08	0.0539	0.4334
BD	4.21	1	4.21	5.08	0.0329	−0.5129
B^2^	5.06	1	5.06	6.1	0.0204	1.2
D^2^	9.07	1	9.07	10.93	0.0028	−1.61
Residual	21.56	26	0.8292			
Lack of Fit	16.34	21	0.778	0.7452	0.7144	
Pure Error	5.22	5	1.04			
Cor Total	48.92	31				

**Table 10:** Predicted optimal processing parameter ranges and the specific conditions selected for the confirmation studies of aqueous cream and cetomacrogol cream. Parameters: **A:** Temperature of oil phase, **B:** Temperature of water phase, **C:** Temperature of stirring environment, **D:** Stirring speed, **E:** End temperature of stirring.

	A (°C)	B (°C)	C (°C)	D (rpm)	E (°C)
Aqueous Cream					
Optimal Values	60–67	76–90	10–15	229–270	48–50
Confirmation study	60	80	10	250	50
Cetomacrogol Cream					
Optimal Values	60–80	75	10–30	216–243	30–50
Confirmation study	70	75	25	220	40

## Data Availability

Data is contained within the article.

## Supplementary Materials

The following supporting information can be downloaded at: https://www.mdpi.com/article/10.3390/pharmaceutics17091232/s1. Figure S1: Summary of the effects of processing conditions on the spreadability, viscosity, and creaming behaviour (Panels B–D) of A: Aqueous creams and B: Cetomacrogol creams. Panel A shows the desirability score that is determined from the combined responses, indicating the processing conditions that are most influential in determining the properties of the final cream. Panels B–D indicate the influence of each processing condition on the respective quality attributes of the final cream (spreadability, viscosity, creaming index); Table S1: Overview of allocated processing condition levels (−1, 0, +1) for the 32 cream samples of aqueous and cetomacrogol creams. Results of the 32 sample creams and their respective commercial counterparts are represented as average responses (mean ± SD, *n* = 3).
