# Supplementary material for: Optimisation of Medicine Compounding Using Quality by Design Approach: Case Studies of Two Aqueous Cream Formulations
Source: Pharmaceutics. 2025 Sep 22;17(9):1232. doi: 10.3390/pharmaceutics17091232 (PMC12473363; doi:10.3390/pharmaceutics17091232)
Supplement: Supplementary file 1 [file pharmaceutics-17-01232-s001.zip › pharmaceutics-3839735-supplementary.pdf]

Supplementary materials

Table S1: Overview of allocated processing condition levels (−1, 0, +1) for the 32 cream samples of aqueous and cetomacrogol creams. Results of the 32 sample creams, and their respective commercial counterparts are represented as average responses (mean ± SD, *n* = 3).

**Sample no.**	**Variables**					**Responses – AQ Creams**			**Responses – CET Creams**		
	**A**	**B**	**C**	**D**	**E**	**Spreadability Ratio**	**Viscosity (mPa·s)**	**Creaming Index (%)**	**Spreadability Ratio**	**Viscosity (mPa·s)**	**Creaming Index (%)**
**10**	1	−1	−1	1	1	1.1 ± 0.2	21,004 ± 146	5.5 ± 2.0	1.1 ± 0.3	54,141 ± 1,268	0 ± 0
**28**	0	0	0	0	0	0.6 ± 0.2	22,997 ± 173	3.3 ± 0.1	1.0 ± 0	76,111 ± 6,894	0 ± 0
**7**	−1	1	1	−1	1	0.2 ± 0.2	3,774 ± 363	93.2 ± 3.4	1.7 ± 0	86,888 ± 4,417	0 ± 0
**30**	0	0	0	0	0	0.4 ± 0	23,362 ± 688	4.4 ± 2.0	0.9 ± 0.1	70,482 ± 3,927	0 ± 0
**32**	0	0	0	0	0	0.7 ± 0.2	22,301 ± 643	4.2 ± 1.8	0.8 ± 0.1	76,675 ± 6,314	0 ± 0
**4**	1	1	−1	−1	1	0.1 ± 0	947 ± 82	93.3 ± 3.3	0.9 ± 0.1	93,645 ± 6,269	0 ± 0
**25**	0	0	0	0	−1	0.6 ± 0.2	23,640 ± 786	44.8 ± 5.4	0.8 ± 0.1	74,398 ± 4,986	0 ± 0
**18**	1	0	0	0	0	0.8 ± 0.3	24,321 ± 708	38.7 ± 5.5	0.7 ± 0.2	65,650 ± 3,822	0 ± 0
**21**	0	0	−1	0	0	1.1 ± 0.3	23,176 ± 671	80.6 ± 16.7	0.6 ± 0.1	84,141 ± 6,623	0 ± 0
**19**	0	−1	0	0	0	0.9 ± 0.1	23,564 ± 890	73.7 ± 17.4	0.8 ± 0.1	54,126 ± 3,469	0 ± 0
**13**	−1	−1	1	1	1	1.4 ± 0.2	22,783 ± 666	75.4 ± 4.2	1.8 ± 0.1	74,318 ± 5,813	0 ± 0
**15**	−1	1	1	1	−1	0.9 ± 0.3	25,993 ± 271	2.2 ± 3.9	0.8 ± 0	68,776 ± 1,364	0 ± 0
**1**	−1	−1	−1	−1	1	0.1 ± 0.2	1,069 ± 130	82.8 ± 2.0	0.7 ± 0.3	43,336 ± 2,823	9.7 ± 0
**6**	1	−1	1	−1	1	0.2 ± 0.1	10,122 ± 346	95.6 ± 2.0	0.7 ± 0.1	52,127 ± 3,369	6.3 ± 0
**12**	1	1	−1	1	−1	1.3 ± 0.3	26,565 ± 1,355	4.2 ± 1.9	0.8 ± 0.1	84,389 ± 8,399	0 ± 0
**9**	−1	−1	−1	1	−1	1.5 ± 0.4	16,004 ± 936	81.7 ± 1.9	1.4 ± 0.1	80,353 ± 7,331	0 ± 0
**26**	0	0	0	0	1	1.1 ± 0.1	19,237 ± 654	7.0 ± 6.5	1.2 ± 0.1	63,037 ± 4,999	0 ± 0
**8**	1	1	1	−1	−1	0.1 ± 0	3,750 ± 158	60 ± 8.8	1.3 ± 0.3	82,507 ± 8,627	0 ± 0
**14**	1	−1	1	1	−1	1.2 ± 0.1	22,384 ± 952	2.3 ± 4.0	1.1 ± 0.1	48,290 ± 4,161	9.4 ± 0
**29**	0	0	0	0	0	1.2 ± 0.1	25,235 ± 712	28.0 ± 4.9	0.6 ± 0.1	64,070 ± 3,333	0 ± 0
**2**	1	−1	−1	−1	−1	0.1 ± 0	567 ± 5	70 ± 5.8	1.0 ± 0.1	46,395 ± 2,034	3.2 ± 0
**23**	0	0	0	−1	0	0.1 ± 0.1	303 ± 15	90.1 ± 5.9	1.1 ± 0.1	60,424 ± 780	3.2 ± 0.1
**22**	0	0	1	0	0	1.1 ± 0.1	25,229 ± 373	20.2 ± 1.4	0.9 ± 0	73,202 ± 2,123	0 ± 0
**11**	−1	1	−1	1	1	1.4 ± 0.1	21,900 ± 933	0 ± 0	0.7 ± 0.1	99,641 ± 4,902	0 ± 0
**17**	−1	0	0	0	0	0.1 ± 0	25,798 ± 187	23.2 ± 1.6	1.1 ± 0.1	67,319 ± 4,388	4.3 ± 1.9
**5**	−1	−1	1	−1	−1	1.8 ± 0.2	3,085 ± 62	69.6 ± 1.7	0.7 ± 0.1	47,234 ± 3,616	8.6 ± 1.9
**31**	0	0	0	0	0	1.8 ± 0.2	23,389 ± 247	3.2 ± 0.1	3.53 ± 1.51	62,833 ± 4,498	2.2 ± 1.9
**20**	0	1	0	0	0	1.5 ± 0.3	28,079 ± 1,299	4.2 ± 1.8	0.8 ± 0.3	97,494 ± 6,384	0 ± 0
**27**	0	0	0	0	0	1.1 ± 0.2	22,113 ± 291	52.7 ± 3.7	0.9 ± 0.4	85,771 ± 5,751	0 ± 0
**3**	−1	1	−1	−1	−1	0.3 ± 0.1	13,009 ± 696	63.4 ± 3.7	2.1 ± 0.1	93,953 ± 6,166	0 ± 0
**16**	1	1	1	1	1	1.8 ± 0.6	34,636 ± 743	0 ± 0	5.19 ± 2.43	103,341 ± 752	0 ± 0
**24**	0	0	0	1	0	2.3 ± 0.1	23,998 ± 657	1.1 ± 1.9	0.3 ± 0.1	29,087 ± 3,461	47.9 ± 0.9
**Commercial**	N/A					1	13,751 ± 1,785	17.2 ±1.9	1	38,214 ± 6,891	21.5 ±1.9

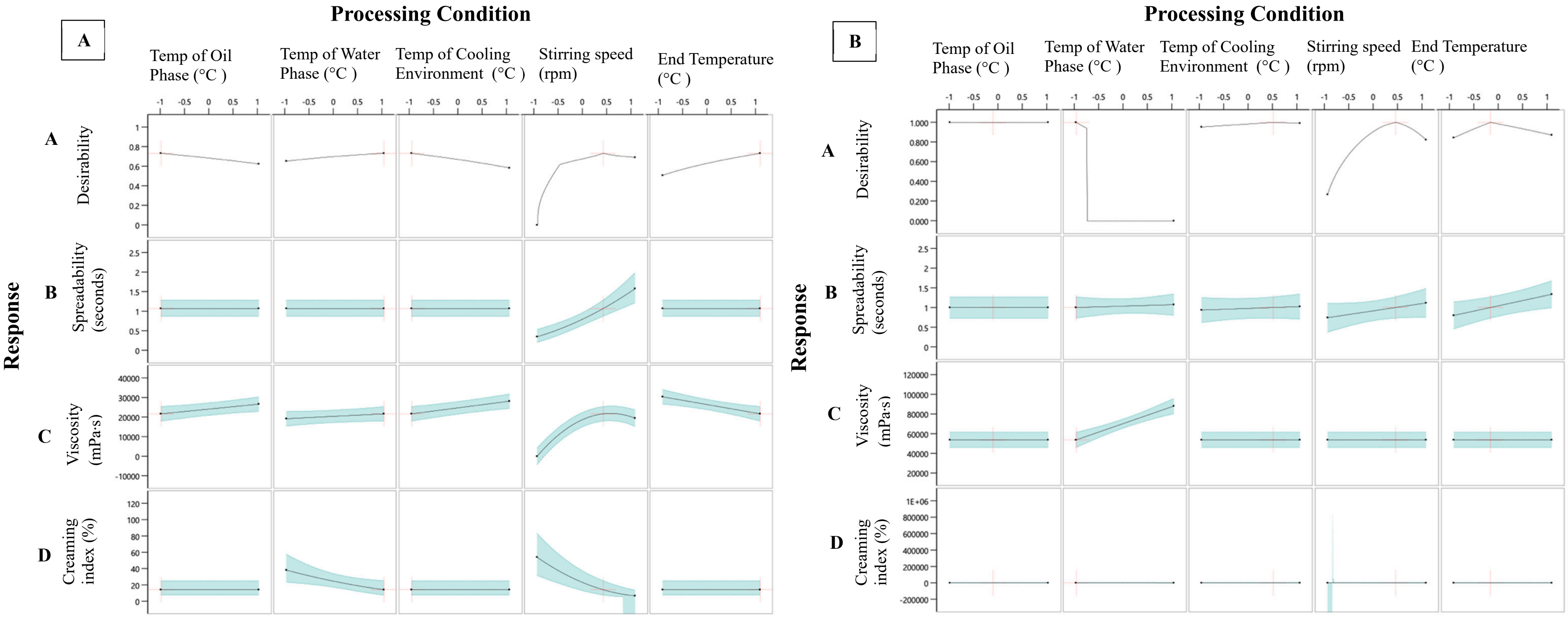

Figure S1: Summary of the effects of processing conditions on the spreadability, viscosity, and creaming behaviour (Panels B – D) of **A:** Aqueous creams and **B:** Cetomacrogol creams. Panel A shows the desirability score that is determined from the combined responses, indicating the processing conditions that are most influential in determining the properties of the final cream. Panels B – D indicate the influence of each processing condition on the respective quality attributes of the final cream (spreadability, viscosity, creaming index).
